# High temperature enhances the ability of *Trichoderma asperellum* to infect *Pleurotus ostreatus* mycelia

**DOI:** 10.1371/journal.pone.0187055

**Published:** 2017-10-26

**Authors:** Zhiheng Qiu, Xiangli Wu, Jinxia Zhang, Chenyang Huang

**Affiliations:** 1 Institute of Agricultural Resources and Regional Planning, Chinese Academy of Agricultural Sciences, Beijing, China; 2 Key Laboratory of Microbial Resources, Ministry of Agriculture, Beijing, China; Universita degli Studi di Pisa, ITALY

## Abstract

*Trichoderma asperellum* is one of the species which can be isolated from contaminated *Pleurotus ostreatus* cultivation substrate with green mold disease. This study focused on the relationship between high temperature and infectivity of *T*. *asperellum* to *P*. *ostreatus*. Antagonism experiments between *T*. *asperellum* and *P*. *ostreatus* mycelia revealed that high temperature-treated *P*. *ostreatus* mycelia were more easily infected by *T*. *asperellum* and covered by conidia. Microscopic observation also showed that *P*. *ostreatus* mycelia treated with high temperature could adsorb more *T*. *asperellum* conidia. Furthermore, conidia obtained from *T*. *asperellum* mycelia grown at 36°C featured higher germination rate compared with that incubated at 28°C. High temperature-treated *T*. *asperellum* mycelia can produce conidia in shorter periods, and *T*. *asperellum* mycelia were less sensitive to high temperature than *P*. *ostreatus*. Deactivated *P*. *ostreatus* mycelia can induce *T*. *asperellum* cell wall-degrading enzymes (CWDEs) and *P*. *ostreatus* mycelia subjected to high temperature showed induced CWDEs more effective than those incubated at 28°C. Moreover, *T*. *asperellum* showed higher CWDEs activity at high temperature. In dual cultures, hydrogen peroxide (H_2_O_2_) increased after 36°C, and high concentration of H_2_O_2_ could significantly inhibit the growth of *P*. *ostreatus* mycelia. In summary, our findings indicated for the first time that high temperature can induce a series of mechanisms to enhance infection abilities of *T*. *asperellum* to *P*. *ostreatus* mycelia and to cause *Pleurotus* green mold disease.

## Introduction

*Pleurotus ostreatus* is one of the most important commercial edible mushroom crop in China. Its production is the third largest among all edible mushrooms in China. *Pleurotus ostreatus* is mainly cultivated using traditional agricultural cultivation methods in which production may be affected due to limited temperature control system. Green mold diseases caused by *Trichoderma* often occur during *P*. *ostreatus* cultivation [1]. These *Trichoderma* spp. mainly include *T*. *pleurotum*, *T*. *pleuroticola*, and *T*. *harzianum* [2,3]. As an emerging problem in *P*. *ostreatus* farms, green mold diseases have resulted in severe crop losses and huge economic losses [4]. Interestingly, extensive green mold diseases frequently occur when *P*. *ostreatus* mycelia have suffered from high temperature in summer during spawn running period. However, little is known about the causes why high temperature-treated *P*. *ostreatus* mycelia are more vulnerable to green molds. Therefore, it is of positive significance to find out the causes of green mold disease.

Green mold diseases caused by *Trichoderma* are characterized by thick green sporulation on substrate surface [3]. *Trichoderma* conidia form quickly and profusely, and can quickly spread in the air due to their low weight. These floating conidia can be adsorbed onto fungal cell wall and recognized by lectin [5]. It was demonstrated that lectin activity plays an important role in host-mycoparasite interaction [6]. Conidia then germinate into mycelia, which quickly occupy nutritional sites and reproduce in mushroom substrate. Temperature is an important abiotic factor that affects *Trichoderma* growth and reproduction. *Trichoderma* conidia produced at high temperature exhibit maximum germination and growth [7,8].

*Trichoderma* spp. have been widely used to control plant pathogenic fungi and improve soil environment [9]. Such biological control includes competition for nutrients and space, production of volatile and nonvolatile antibiotics, and production of hydrolytic enzymes [10]. However, *Trichoderma* spp. are common pathogenic fungi for edible mushrooms [11]. *Trichoderma* spp. can grow on the mushroom beds and finally cover the entire substrate. *Trichoderma* features a variety of mechanisms that inhibit or degrade other fungal mycelia [12]. To infect other fungal cells, *Trichoderma* species should first recognize the host cell wall, coil around its mycelia, develop appressoria, and then secrete cell wall degrading enzymes (CWDEs) [13]. *Trichoderma* species can produce a wide variety of CWDEs, such as chitinases, β-1,3-glucanases and proteases to hydrolyze the main components of host cell wall during antagonism [14], which is a main mechanism for controlling other fungi [15]. In the presence of host fungi, production and enzyme activity of these CWDEs secreted by *Trichoderma* could be further induced [16].

Previous studies have focused on isolation and identification of pathogenic fungi causing *Pleurotus* green mold diseases [17,18]. *Trichoderma asperellum* has been reported as one of the species in *Pleurotus* substrates with green mold disease [3]. Studies showed that *P*. *ostreatus* substrates subjected to high temperature were more easily infected by *Trichoderma* during *P*. *ostreatus* cultivation. The aim of this study is to investigate the effects of high temperature on *T*. *asperellum* infecting *P*. *ostreatus* mycelium and find out the relationship between high temperature and rampant breakout of *T*. *asperellum* in *P*. *ostreatus* spawn running stage.

## Materials and methods

### Strains, media and growth conditions

*Pleurotus ostreatus* P89 (CCMSSC 00389) was provided by the China Center for Mushroom Spawn Standards and Control.

*Trichoderma asperellum* T11 (ACCC 32725) was deposited at the Agricultural Culture Collection of China. The internal transcribed spacer region (ITS) sequence was amplified by the primers ITS1 (5’-TCCGTAGGTGAACCTGCGG-3’) and ITS4 (5’-TCCTCCGCTTATTGATATGC-3’), and translation elongation factor 1α (*tef*1) sequence was amplified by primers EF1-728F (5’-CATCGAGAAGTTCGAGAAGG-3’) and TEF-LLErev (5’-AACTTGCAGGCAATGTGG-3’) [3]. ITS and *tef1* sequences of *T*. *asperellum* T11 were deposited in NCBI GenBank (KY368169 and MF049065)

In order to verify that *T*. *asperellum* can cause *Pleurotus* green mold disease, Koch's Postulates were applied (S1 Fig). Ten pieces of P89 mycelial discs (5 mm diameter) were inoculated into cotton seed hull substrate and incubated at 28°C for 10 days. Afterward, *P*. *ostreatus* bags were respectively placed in incubators with different constant temperature at 28°C and 36°C for 2 days. Finally, five pieces of T11 discs were inoculated into the bags and incubated at 28°C. Each trail consisted of 5 bags.

For enzyme assay, conidial suspension collected from strain T11 was inoculated into 250 mL Erlenmeyer flasks containing 100 mL of liquid minimal synthetic medium (MSM). One liter of MSM contained 5 g glucose, 0.2 g MgSO_4_·7H_2_O, 0.9 g K_2_HPO_4_, 0.2 g KCl, 1.0 g NH_4_NO_3_, 0.002 g FeSO_4_·7H_2_O, 0.002 g MnSO_4_, 0.002 g ZnSO_4_ and distilled water [19]. For other experiments, T11 and P89 mycelia were cultured on potato dextrose agar (PDA; Difco-Becton Dickinson, Sparks, MD) and potato dextrose broth (PDB; Difco-Becton Dickinson, Sparks, MD). To cover the whole plate, mycelia of T11 and P89 were grown on PDA at 28°C for 3 and 7 days, respectively.

### Collection of deactivated *P*. *ostreatus* mycelia

Fifteen pieces (a diameter of 5 mm punch) of P89 mycelial discs from solid medium were inoculated into 300 mL of PDB and incubated in a shaking incubator at 150 rpm and 28°C for 5 days. Then, the Erlenmeyer flasks were divided equally into four parts and incubated at 28°C, 32°C, 36°C and 40°C respectively for 2 days. Finally, mycelia were collected with filter paper (Whatman, Ф11), washed thrice with distilled water, dried under vacuum and grounded into powder [20].

### Antagonism experiments between *T*. *asperellum* and *P*. *ostreatus* mycelia

P89 mycelial discs (5 mm diameter) were placed at one side of PDA plates and incubated at 28°C for 3 days. Then, the plates were respectively placed in incubators with different constant temperatures of 28°C, 32°C, 36°C and 40°C for 2 days. Then, T11 mycelial discs were inoculated at the other side of plates and incubated at 28°C for 7 days [21,22]. The distance between two mycelial discs measured 5 cm. All tests were performed in triplicate.

### Agglutination reaction between *T*. *asperellum* conidia and *P*. *ostreatus* mycelia

To examine agglutination between *T*. *asperellum* conidia and *P*. *ostreatus* mycelia, P89 was grown on a thin layer of PDA on microscope slides at 28°C for 3 days. Then, the plates were transferred to incubators with different temperature at 28°C and 36°C for 2 days. Conidial suspension (1.86×10^7^ CFU/mL) in phosphate buffered saline (PBS) was used to observe agglutination reaction. Attachment of T11 conidia to P89 mycelia was observed in dual culture. Conidial suspension was poured over microscope slides with P89 mycelia. Reaction was carried at 28°C for 30 min. Then, the suspension was gently washed thrice with distilled water. Finally, attachment of T11 conidia to P89 mycelia was observed under light microscopes. An optical microscope (OLYMPUS DP70, Tokyo, Japan) was used for observations. The magnification was 400 times.

### Determination of *T*. *asperellum* conidial germination rate after high temperature treatment

T11 disc was inoculated on PDA and incubated at 28°C for 10 days. Then, T11 plates with numerous of conidia were transferred to incubator at 36°C for various periods of time (2 h, 6 h, 12 h, 24 h, and 48 h). Conidia were harvested separately with SDW. Conidial numbers in suspension were determined using a hemocytometer and adjusted to appropriate concentration. Then, 100 μL of diluted conidium suspension was spread onto 2% water agar plates [23]. All plates were incubated at 28°C for 2 days, then the number of germinated conidia was recorded. Each treatment group consisted of three replicates.

### Conidium formation time after high temperature treatments for different times

T11 discs were inoculated on the center of plates and incubated at 28°C for 3 days. Plates were incubated at 36°C for different times (2 h, 4 h, 6 h, 8 h, 12 h, 24 h, 48 h, and 72 h) and then transferred to 28°C to observe formation of conidia. Conidium formation time was denoted as the time when the whole plate was covered with green conidia.

### Mycelial growth at different temperatures

Both P89 and T11mycelial discs were inoculated on the center of PDA plates and incubated at 28°C. When colony diameter reached 1 cm, the plates were transferred into different incubators at 28°C, 32°C, 36°C, and 40°C, respectively. Then, colony diameter in each plate was recorded daily [24].

### Enzymatic assays

For enzymatic assays, T11 conidia were inoculated into 100 mL MSM and MSMP. The formula of MSMP was exactly the same as MSM except that 5 g glucose was replaced by 3 g glucose and 2 g deactivated *P*. *ostreatus* mycelia. To detect the effect of temperature on enzyme activity, T11 conidia (1.80×10^7^ CFU/mL) were inoculated in MSM and MSMP (using deactivated *P*. *ostreatus* mycelia cultivated at 28°C), and cultures were incubated for 3 days at 28°C with shaking at 150 rpm. Then, flasks were transferred to rotary shakers (ZQLY-180F, Shanghai Zhichu Instrument Company Limited, Shanghai, China) with different temperature (28°C, 32°C, 36°C, and 40°C) for 2 days. Liquid culture was collected by centrifuging (Sigma 3K30, Osterode, German) at 6,000×g for 10 min and supernatants were used as crude enzyme solutions or stored at -80°C until assayed. To detect the effect of different substrates on enzyme activity, T11 conidia (1.58×10^7^ CFU/mL) were inoculated in MSMP, which used deactivated *P*. *ostreatus* mycelia collected from cultures growing at different temperatures. Cultures were incubated for 3 days at 28°C with shaking at 150 rpm. Then, crude enzyme solutions were collected as above.

Chitinase activity was determined by the method of dinitrosalicylic acid (DNS) [25]. The reaction mixture contained 0.1 mL of 1% (w/v) colloidal chitin in 10 mM phosphate buffer (pH 7.5) and 0.1 mL of crude enzyme solution. Colloidal chitin (1%) was prepared according to the method of Lee et al. [26]. First, reaction solution was mixed and incubated at 40°C for 30 min, reaction was stopped by boiling for 3 min, cooled to 25°C for 2 min and centrifuged (Sigma 3K30, Osterode, German) at 10,000×g for 5 min. The supernatant with 100 μL volume was mixed with equal volume of DNS, and heated to 100°C for 10 min. Absorbance was measured at 540 nm in Thermo Scientific Microplate Reader (Scientific Fluoroskan Ascent FL, Waltham, MA, USA). One unit (U) of enzyme activity was defined as the amount of enzyme to release 1μmol of N-acetyl-glucosamine in reaction mixture per min, whereas GlcNAc (Sigma) was used as the standard.

Laminarin (Sigma) (0.75% w/v) in 50 mM sodium acetate buffer (pH 5.0) was used to determine β-1,3-glucanase activity [27]. The reaction mixture contained 20 μL laminarin solution and 10 μL of crude enzyme solution in a microplate. The mixture was incubated at 50°C for 10 min. Afterward, 100 μL of DNS reagent was added to mixture, heated to 95°C for 5 min, and then cooled to 25°C for 2 min. The reaction mixture (100 μL) was transferred to an enzyme-linked immunosorbent assay (ELISA) microplate and absorbance was measured at 540 nm in Thermo Scientific Microplate Reader. One unit (U) of enzyme activity was defined as the amount of enzyme required to release 1 μmol of reducing sugar per minute.

Protease activities were determined using casein as substrate and assayed according to the method of Satake et al. [28] with slight modification. The assay mixture contained 100 μL of crude enzyme solution and 200 μL of casein solution. Assay mixture was incubated at 37°C for 1 h. Subsequently, 400 μL of 5% trichloroacetic acid (TCA) was added to stop the reaction. The solution was centrifuged at 12,000×g for 5 min at 4°C. Absorbance of supernatant was measured at 280 nm using a UV-spectrophotometer (TU-1810, PERSEE, Beijing, China). One unit (U) of protease activity was defined as a 0.001 absorbance increase per minute in supernatant per milliliter of reaction mixture [29,30].

### Detection of hydrogen peroxide accumulation in dual cultures of *T*. *asperellum* and *P*. *ostreatus* under high temperature

To detect H_2_O_2_ accumulation in antagonism between *T*. *asperellum* and *P*. *ostreatus*, T11 and P89 were evaluated in dual culture tests. P89 mycelia were inoculated into 250 mL Erlenmeyer flasks containing 100 mL PDB and incubated for 3 days at 28°C with shaking at 150 rpm. Then, 1 mL of T11 conidial suspension (1.69×10^7^ CFU/mL) was inoculated into flasks containing P89 mycelia and incubated at 28°C for 1 day. Then, flasks were transferred to rotary shakers (ZQLY-180F, Shanghai Zhichu Instrument Company Limited, Shanghai, China) at 28°C and 36°C, respectively for different time (1 h, 2 h, 6 h, 12 h, 24 h and 48 h). PDB flasks inoculated with either T11 only or P89 only were used as controls at 28°C for 3 days. Extracellular fluid was collected by centrifugation for 5 min at 8,000 rpm. The H_2_O_2_ content was determined using a Hydrogen Peroxide Assay Kit (Nanjing Jiancheng Bioengineering Institute, Nanjing, China), following the manufacturer’s instructions. Absorbance was measured at 405 nm.

### Sensitivity of mycelia to H_2_O_2_

High concentration of H_2_O_2_ can inhibit the growth of mycelium. In order to assess sensitivity of *T*. *asperellum* and *P*. *ostreatus* mycelia to H_2_O_2_, P89 mycelial discs and T11 mycelial discs were inoculated on the center of PDA plates with different concentrations of H_2_O_2_ and incubated at 28°C. When the majority of plates were occupied by mycelia, diameter and morphology of colonies were observed.

### Statistical analysis

Analysis of variance (ANOVA) and Duncan’s multiple range tests (*P* ≤ 0.05) were applied to calculate and analyze data. Data were analyzed using SPSS version 20.0 software (SPSS Inc., Chicago, IL, USA). Error bars represent the standard deviation of three replicates.

## Results

### Antagonism of *T*. *asperellum* against *P*. *ostreatus* after high temperature treatment

**Fig 1:**
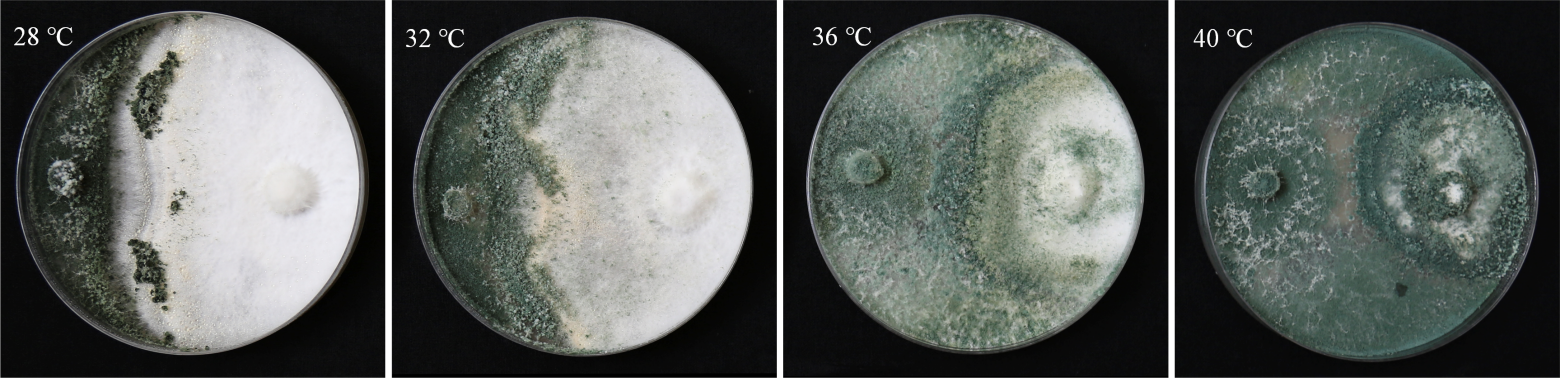
Overgrowth and growth inhibition of *T*. *asperellum* and *P*. *ostreatus*. Each plate contained *T*. *asperellum* at the left and *P*. *ostreatus* at the right. *Pleurotus ostreatus* mycelia were first placed at one side and incubated at 28°C for 3 days and then treated with different temperatures (28°C, 32°C, 36°C and 40°C) for 2 days. *Trichoderma asperellum* mycelia were then inoculated at opposite sides and incubated at 28°C for 7 days.

To observe infection of *T*. *asperellum* to high temperature-treated *P*. *ostreatus* mycelia, the interaction between *T*. *asperellum* and *P*. *ostreatus* was investigated by using a dual culture test in the plates. After various temperature treatments, P89 mycelia resisted infection of T11 at different degrees. T11 was able to overgrow and sporulate over P89 mycelia. P89 mycelia grown at 28°C showed stronger resistance to T11 than those treated with 36°C and 40°C. After incubation at 36°C and 40°C, P89 mycelia were almost completely inhibited by T11 mycelia and covered by T11 conidia (Fig 1). By contrast, only a few mycelia on the edge of plates incubated at 28°C were covered by T11 conidia. It indicated that high temperature will reduce resistance of *P*. *ostreatus* mycelia to *T*. *asperellum*.

### Agglutination reaction between *T*. *asperellum* conidia and *P*. *ostreatus* mycelia

**Fig 2:**
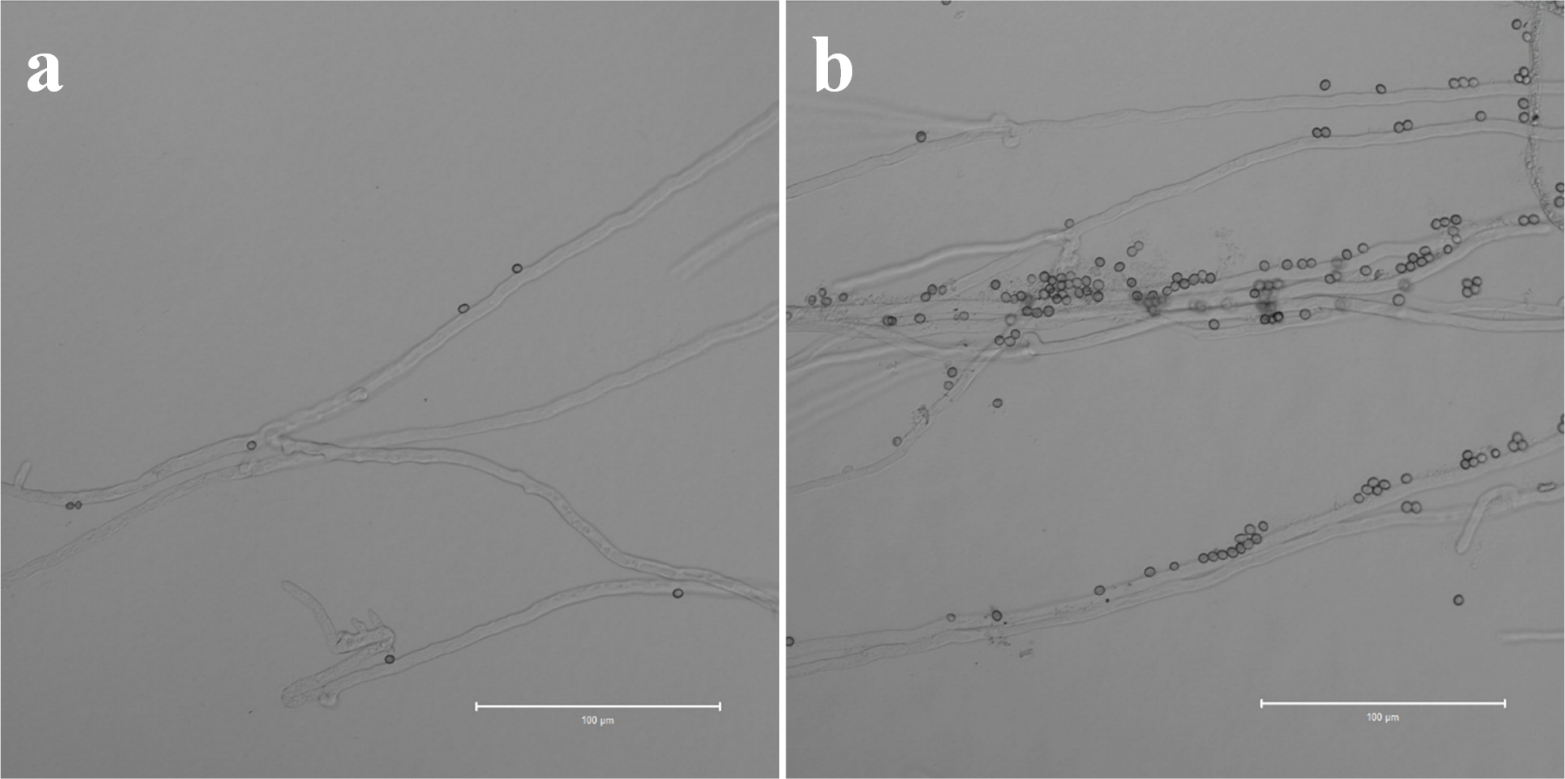
Attachment of *T*. *asperellum* conidia to *P*. *ostreatus* mycelia (bar = 100 μm, ×400). **a** Agglutination reaction between conidia to *P*. *ostreatus* grown at 28°C. **b** Agglutination reaction between conidia and *P*. *ostreatus* mycelia which were subjected to 36°C for 2 days.

Attachment of T11 conidia to P89 mycelia was observed under light microscopes. P89 mycelia incubated at various temperature showed different adsorption ability to T11 conidia. Only a few T11 conidia were adsorbed on the surface of mycelia incubated at 28°C (Fig 2A). P89 mycelia dealt with 36°C for 2 days can adsorb more spores on its surface, and dozens or more conidia were observed on mycelia in each field of vision (Fig 2B). P89 mycelia affected by high temperature were more prone to adsorb free T11 conidia.

### Effect of high temperature on germination rate of *T*. *asperellum* conidia

**Fig 3:**
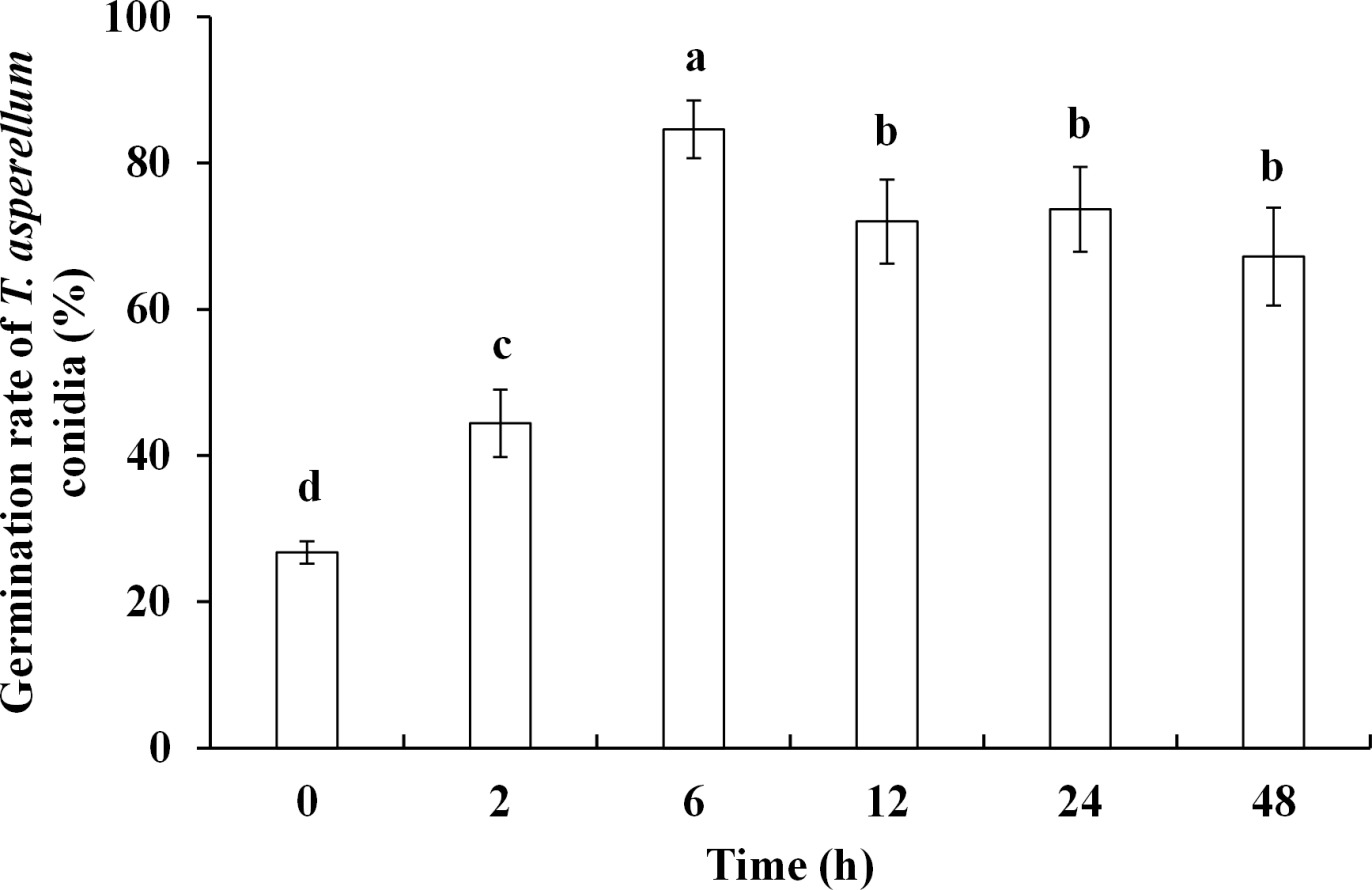
Germination rate of *T*. *asperellum* conidia. *Trichoderma asperellum* conidia were collected from plates treated at 36°C for different times. Data were analyzed by Duncan’s ANOVA test. Error bars represent the standard deviation of three replicates.

Conidia germination is an important critical step in *Trichoderma* infection. T11 conidia harvested at 36°C presented significantly (*P* ≤ 0.05) higher germination rates than those at 28°C. Heat treatment time at 36°C can also affect conidia germination rates. Germination rates increased sharply upon heat treatment and reached the highest level at 6 h, then declined gradually and remained stable at high levels until 48 h (Fig 3). This result indicated that T11 conidia germination was affected by temperature, and conidia subjected to high temperature exhibited higher germination rate.

### Effect of high temperature on *T*. *asperellum* sporulation

**Fig 4:**
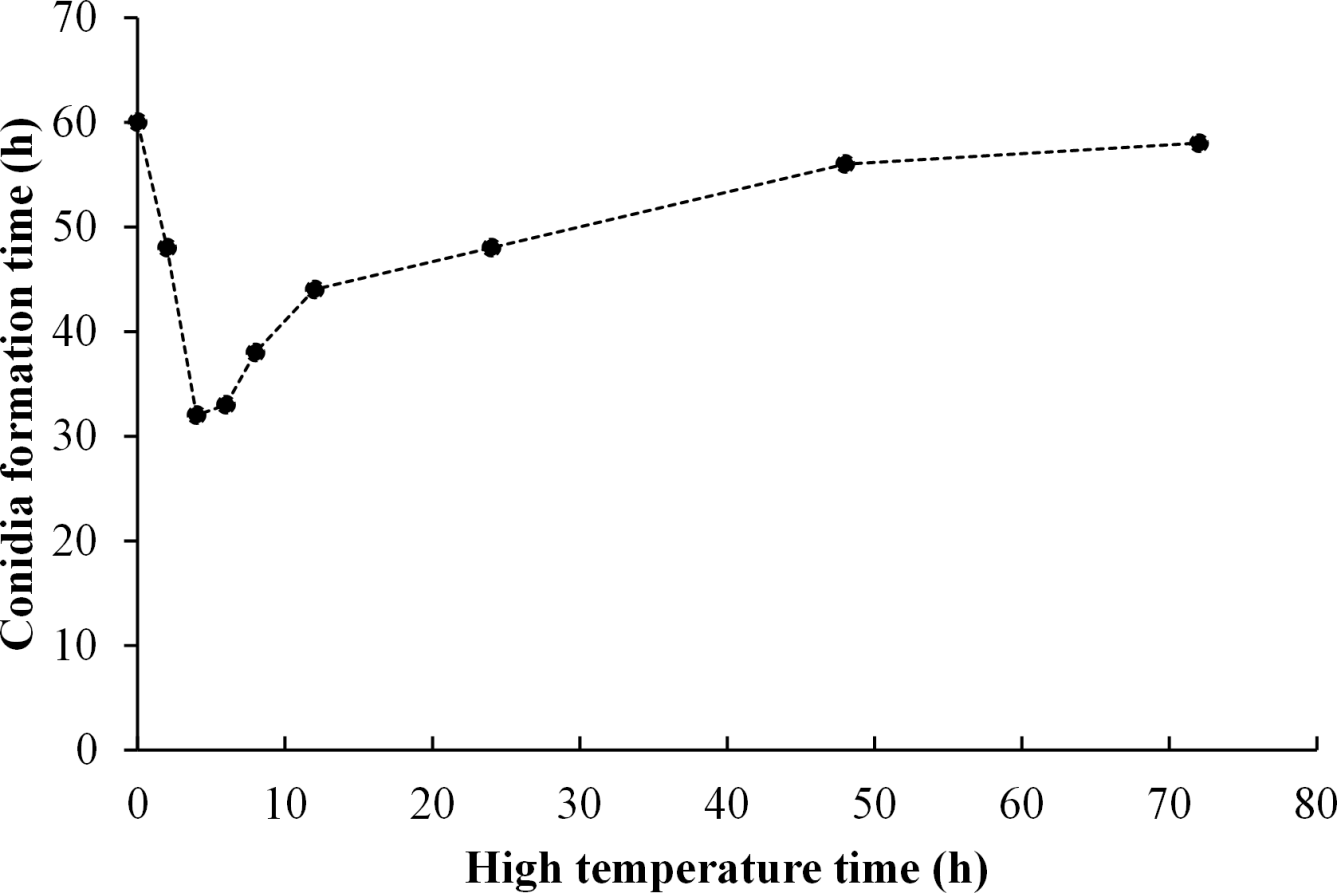
Conidium formation time after high temperature treatment.

Temperature and incubation time affected formation of T11 conidia. A strong temperature effect was observed, and after incubating mycelia at 36°C for different time periods, conidium formation time was shortened generally (Fig 4). Conidium formation time decreased abruptly during early heat treatment period, reduced to minimum at 4 h, and then kept rising from 6 h and almost reached the original level in the end. In conclusion, high temperature accelerated T11 sporulation especially during short treatment period. As heat treating time extended, the acceleration effect became negligible.

### Effect of temperature on mycelial growth

**Fig 5:**
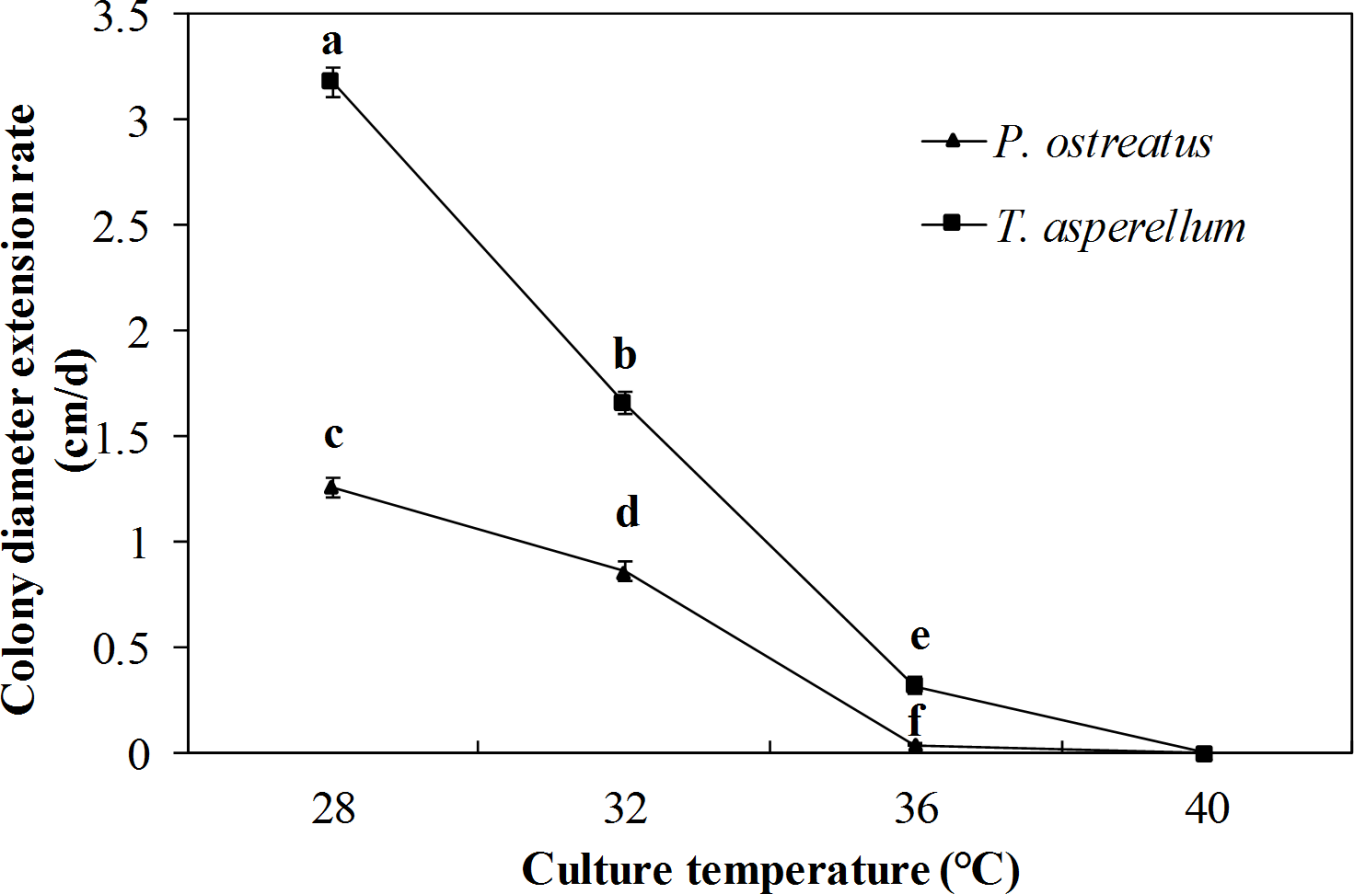
Effect of temperature on mycelial growth of *T*. *asperellum* and *P*. *ostreatus*. Data were analyzed by Duncan’s ANOVA test. Error bars represent the standard deviation of three replicates.

To determine the effect of temperature on *T*. *asperellum* and *P*. *ostreatus* mycelial growth, mycelial discs of T11 and P89 were inoculated on the center of PDA plates and cultivated at different temperature conditions (28°C, 32°C, 36°C, and 40°C). Growth trends of T11 and P89 mycelia in response to temperature were similar. As temperature elevated, colony diameter denoting growth rate of both strains declined significantly. T11 mycelia grew significantly (*P* ≤ 0.05) faster than P89 at 28°C, 32°C and 36°C (Fig 5). T11 slowly grew at 36°C, whereas P89 was almost completely suppressed. Neither T11 nor P89 mycelia can grow at 40°C (Fig 5). These results revealed that *T*. *asperellum* and *P*. *ostreatus* mycelia were affected by temperature and *P*. *ostreatus* mycelia were more sensitive to high temperatures than *T*. *asperellum*.

### Effect of temperature on enzyme activities with or without deactivated *P*. *ostreatus* mycelia

**Fig 6:**
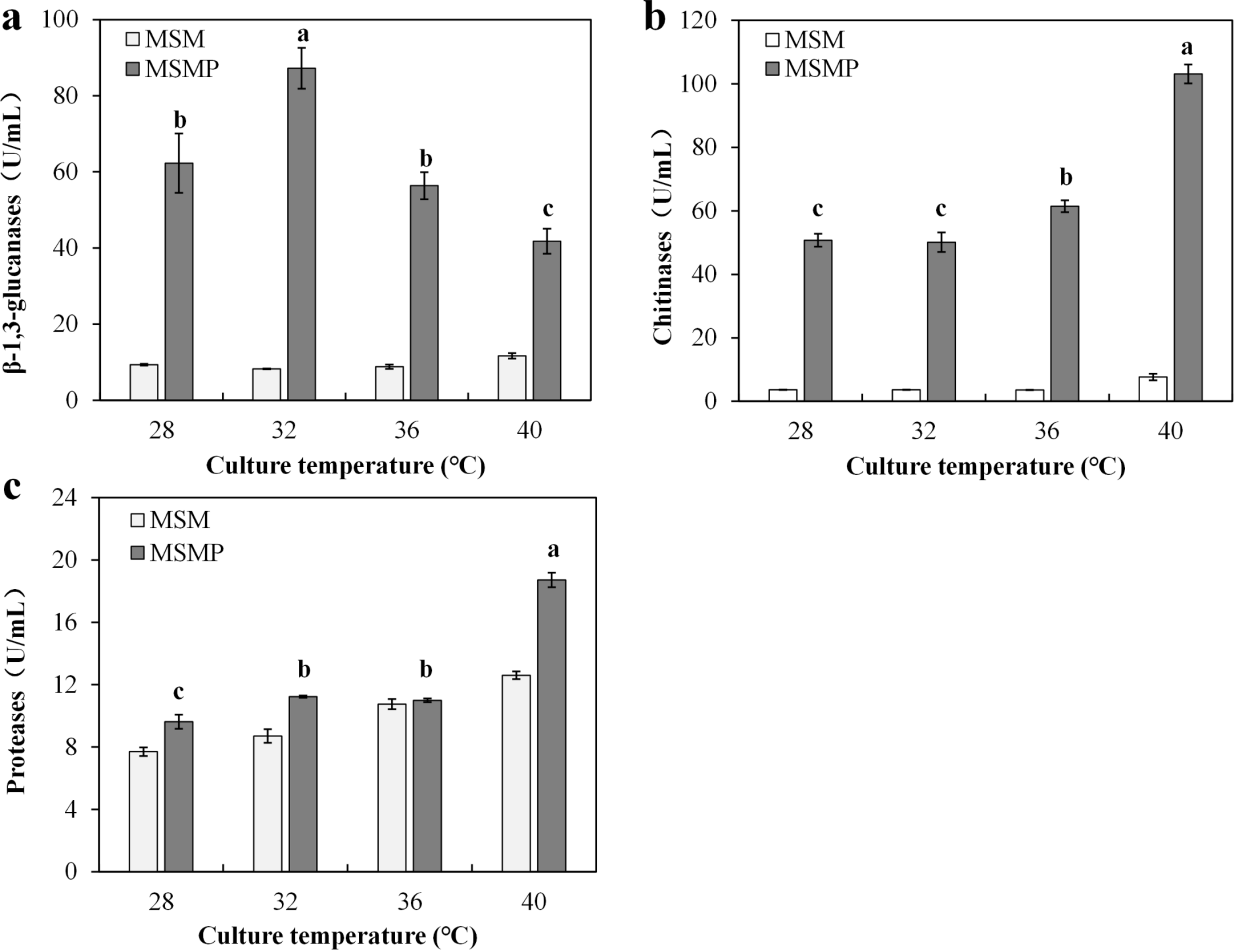
*Trichoderma asperellum* extracellular enzyme activity affected by different temperatures. Effect of temperature on β-1,3-glucanases (**a**), chitinase (**b**), protease (**c**) in two media with or without deactivated *P*. *ostreatus* mycelia. Data were analyzed by Duncan’s ANOVA test. Error bars represent the standard deviation of three replicates.

In general, *T*. *asperellum* was capable of secreting a number of CWDEs, including β-1,3-glucanases, chitinases and proteases. Overall, in the presence of deactivated *P*. *ostreatus* mycelia, activities of CWDEs were almost higher than those incubated in MSM. Moreover, high temperature also showed significant effects on CWDEs activity. For β-1,3-glucanases, the highest specific activities (87.20 U/mL) were exhibited at 32°C (Fig 6A). For chitinase and protease, the maximum activities (103.11 U/mL and 18.71 U/mL, respectively) were detected at 40°C, and enzyme activities at 36°C were also significantly (*P* ≤ 0.05) higher than that at 28°C (Fig 6B and 6C). It was shown that deactivated *P*. *ostreatus* mycelia and high temperature can separately or jointly induce higher activities of CWDEs, which are conducive to hydrolysis of *P*. *ostreatus* cell wall.

### Effect of substrates on *T*. *asperellum* enzyme activities

**Fig 7:**
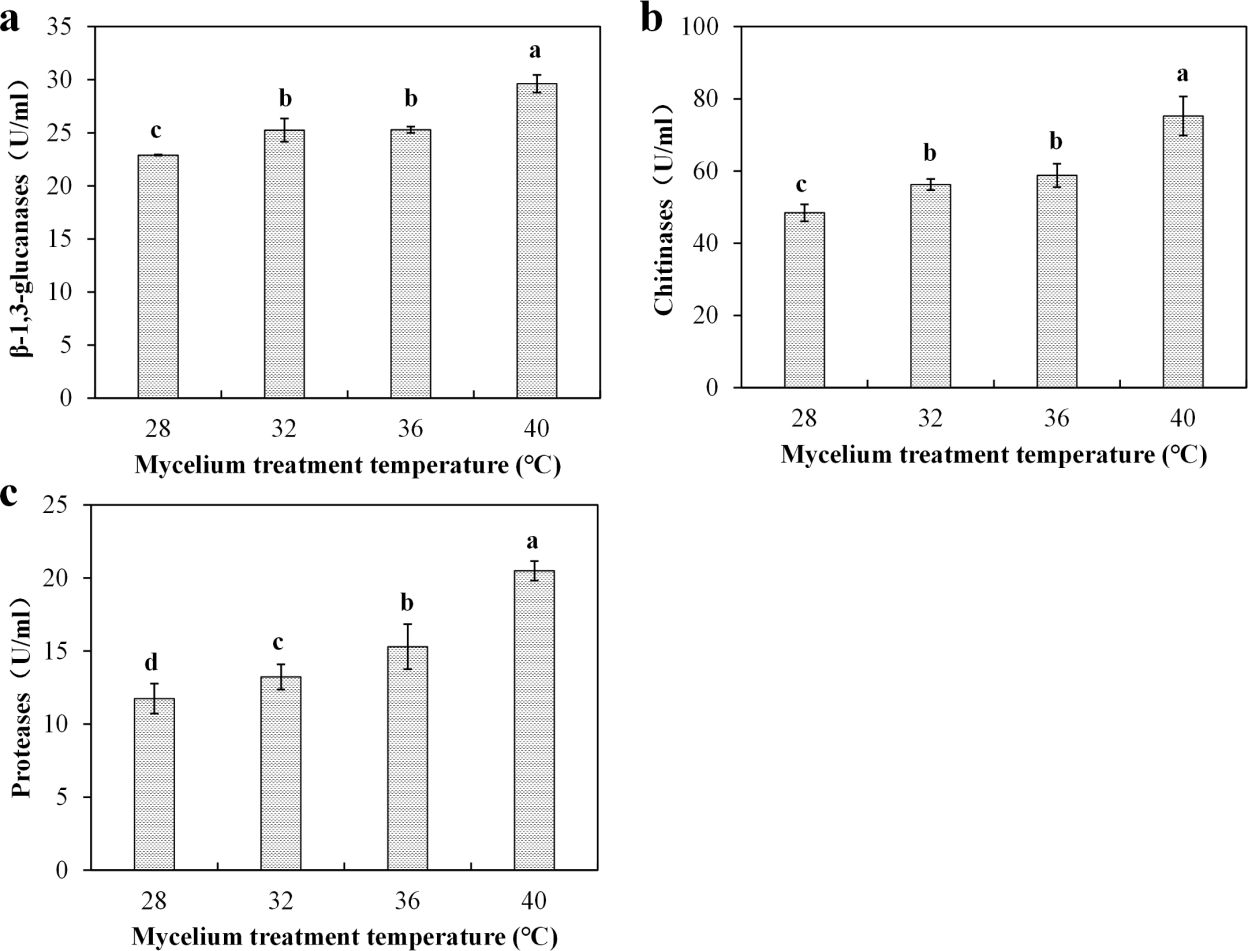
*Trichoderma asperellum* extracellular enzyme activity is affected by different substrates. Effect of different substrates on β-1,3-glucanases (**a**), chitinase (**b**), protease (**c**). Data were analyzed by Duncan’s ANOVA test. Error bars represent the standard deviation of three replicates.

Deactivated *P*. *ostreatus* mycelia collected from different temperature conditions were used to assay CWDE activity. P89 mycelia treated with high temperature (32°C, 36°C and 40°C) induced enzyme activities of β-1,3-glucanases, chitinases and proteases, and their enzyme activities were significantly (*P* ≤ 0.05) higher than those cultivated with *P*. *ostreatus* mycelia collected from 28°C (Fig 7). For all enzymes, the maximum activities were observed in cultures with mycelia collected from 40°C (Fig 7). Analysis of enzyme activities suggested that *T*. *asperellum* CWDEs activity was further induced by *P*. *ostreatus* mycelia subjected to high temperatures.

### Effect of temperature on H_2_O_2_ content and effect of H_2_O_2_ on mycelial growth of *T*. *asperellum* and *P*. *ostreatus*

**Fig 8:**
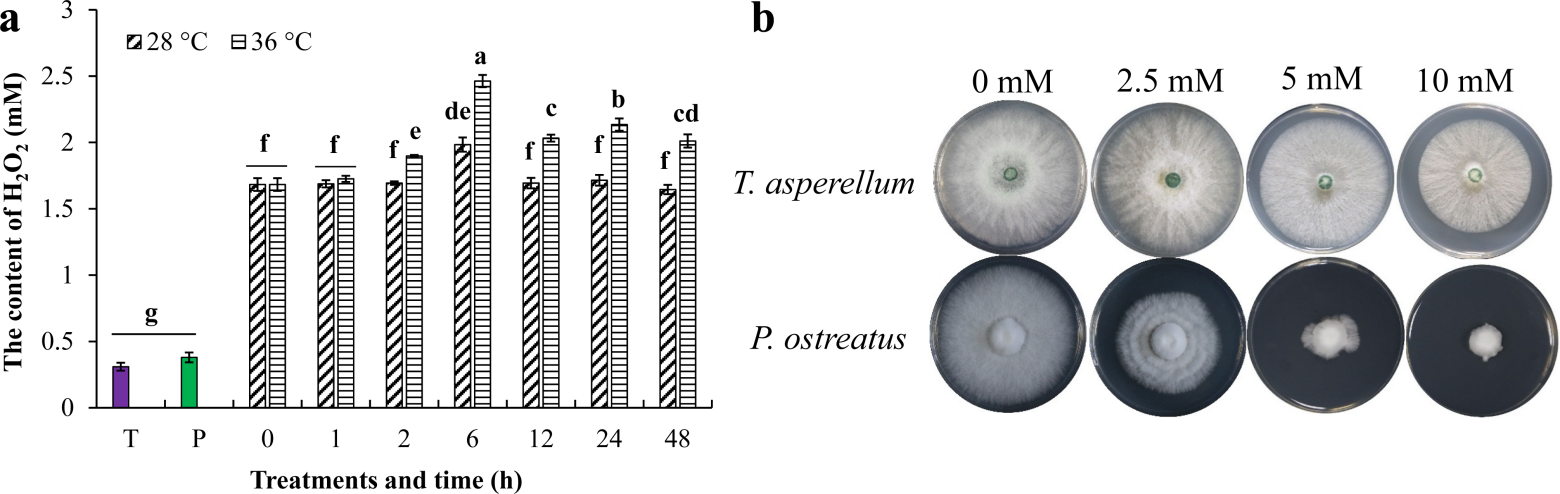
Accumulation of H_2_O_2_ in dual cultures and effect of different concentrations of H_2_O_2_ on growth inhibition of mycelia. **a** High amounts of H_2_O_2_ accumulated in dual culture, and high temperature can significantly promote H_2_O_2_ accumulation at 36°C and reach the highest at 36°C for 6 h. T: PDB was inoculated with *T*. *asperellum* only. P: PDB was inoculated with *P*. *ostreatus* only. **b** A total of 2.5 mM H_2_O_2_ can significantly inhibit growth of *P*. *ostreatus* mycelia, whereas *T*. *asperellum* mycelium was not sensitive to H_2_O_2_.

Extracellular H_2_O_2_ content of *T*. *asperellum* only, *P*. *ostreatus* only and dual cultures were detected. H_2_O_2_ content in cultures with T11 mycelia only (0.31 mM) and P89 mycelia only (0.38 mM) were significantly (*P* ≤ 0.05) less than that in dual cultures (Fig 8A). H_2_O_2_ content significantly increased when dual cultures were cultivated at 36°C from 2–48 h compared with those at 28°C. These results revealed that co-cultivation of both strains will stimulate generation of H_2_O_2_ which was generally accepted as detrimental to cell growth. H_2_O_2_ content increased to the highest level at 36°C for 6 h (2.46 mM). Thus, effects of various concentration of H_2_O_2_ on T11 and P89 mycelial growth were detected. Results showed that H_2_O_2_ over 2.5 mM can significantly inhibit growth of *P*. *ostreatus* mycelium, and 10 mM of H_2_O_2_ almost completely suppressed the growth of P89 mycelium (Fig 8B). T11 mycelia were not sensitive to low concentration of H_2_O_2_ and grew normally in 2.5 mM, whereas 5 mM H_2_O_2_ can only slightly inhibit growth of T11 mycelia (S2 Fig). These results revealed that high temperature induced production of H_2_O_2_ in dual culture, and high concentration of H_2_O_2_ severely inhibited *P*. *ostreatus* mycelial growth rather than *T*. *asperellum*.

## Discussion

Different *Trichoderma* species live in different environments, and different climatic conditions determine their distributions. Many *Trichoderma* species have been isolated from infected oyster mushroom substrates [31,32]. In our study, *T*. *asperellum* was one of the most common strains in green molded substrate. Optimal growth temperature for *Pleurotus* spp. mycelia measures 20–30°C, and mycelial growth will decline when temperature increases up to 30°C [33,34]. For *Trichoderma* spp., optimal temperature spans 27–30°C [35]. The present study provides a new viewpoint that high temperature may be an important factor to cause the green mold diseases.

Other studies have observed antagonism by *Trichoderma* against other fungi [21,36]. In our study, *T*. *asperellum* can outgrow *P*. *ostreatus* mycelia, produce more conidia and significantly inhibit growth of *P*. *ostreatus* mycelia after they suffered from high temperature (Fig 1). In addition, *P*. *ostreatus* mycelia subjected to high temperature were infected by *T*. *asperellum* (S1 Fig). These phenomena may demonstrate that the resistance of *P*. *ostreatus* mycelia to *T*. *asperellum* severely decreased after high temperature treatment (36°C, 40°C) or the ability of *T*. *asperellum* to infect *P*. *ostreatus* mycelium enhanced.

Mycoparasitism has been considered the central mechanism for *Trichoderma* infection in other fungi. Recognition is a prerequisite in inhibiting and hydrolyzing other fungal cells. *Trichoderma asperellum* attaches to other fungal cell with cell wall carbohydrates that bind to fungal lectins [37]. In the present study, we observed that *P*. *ostreatus* mycelia treated at 36°C for 2 days can adsorb a large number of conidia (Fig 2), and this result had not been observed before. This phenomenon suggests that high temperature may increase the lectin content of *P*. *ostreatus* cell wall, causing *P*. *ostreatus* mycelia to adsorb more conidia and be infected by *T*. *asperellum*. This result also explains why hot summer weather possibly particularly causes outbreaks of green mold disease.

*Trichoderma asperellum* conidia will germinate to form filamentous hyphae after attaching to *P*. *ostreatus* mycelia. In our study, germination rate of conidia showed an increasing trend after high temperature-treatment for different time periods. Conidia collected at 36°C gave the greater germination in comparison with that at 28°C. Previous publications confirmed that *Trichoderma* conidia can accumulate intracellular polyols such as trehalose and mannitol under high temperatures. These substances can increase germination rate, enhance conidial bioactivity and tolerance to adverse environmental conditions [38,39]. We discovered that *T*. *asperellum* mycelia treated with high temperature (36°C) can produce conidia in shorter periods compared with that incubated at 28°C (Fig 4). Possibly, high temperature may accelerate the transition from *T*. *asperellum* mycelial vegetative growth to reproductive growth. Thus, high temperature can cause *T*. *asperellum* mycelia to produce a large number of conidia in a very short period of time and speed up the spread of conidia, resulting in a large area of affected by green mold disease.

Temperature is an important environmental factor during mushroom mycelial growth. In the present study, the results showed that *P*. *ostreatus* mycelia were more sensitive than *T*. *asperellum* to high temperature. Growth of *P*. *ostreatus* mycelia was almost completely suppressed at 36°C (Fig 5). At the same time, mycelium growth of *T*. *asperellum* was significantly faster than that of *P*. *ostreatus* at 28°C, 32°C and 36°C. The rapid growth of *T*. *asperellum* is conductive to its occupation of nutritional sites and space [40].

During the mycoparasitism, extracellular CDWEs secreted by *Trichoderma* can destroy other fungal cell walls [41]. In the present study, CDWE activities produced in MSMP were higher than those produced in MSM (Fig 6), demonstrating that deactivated *P*. *ostreatus* cell can promote extracellular enzyme activity. Zhang et al. [16] also reported that deactivated *Fusarium oxysporum* mycelia were more effective in inducing production and activities of *T*. *harzianum* extracellular enzymes. We also noted that high temperature can enhance extracellular enzyme activities. The highest activities for β-1,3-glucanases were detected at 32°C, whereas those for chitinase and protease were observed at 40°C. Previous studies have also found that found that the optimum temperature for chitinase was 40°C [42,43]. These findings can explain that high temperature can enhance the ability of *T*. *asperellum* to hydrolyze *P*. *ostreatus* cell wall. At the same time, *Trichoderma* CWDEs are inducible by substrate [44]. In this study, we investigated the effects of different substrates on enzyme activity (Fig 7). The results showed that *P*. *ostreatus* mycelia harvested at high temperature can significantly induce enzyme activity. The findings are similar to those in a previous study, which showed that CWDEs are influenced by substrates [45,46]. According to the results, we infer that high temperature may change intracellular metabolites of *P*. *ostreatus* mycelia or cell wall components and these changes can induce *T*. *asperellum* extracellular enzyme activity.

During antagonism, the fungal cell can induce synthesis of reactive oxygen species (ROS), such as hydrogen peroxide (H_2_O_2_) and superoxide radicals (O_2_^.-^), during their direct confrontation by *Trichoderma*, and excessive ROS levels can cause cell death, damage cell membrane and increase membrane permeability, and cause electrolyte leakage [47–49]. In the present study, extremely low concentration of extracellular H_2_O_2_ was detected in cultures with either *T*. *asperellum* only or *P*. *ostreatus* only; however, high concentration of H_2_O_2_ accumulated in dual cultures, and high temperature can further promote the accumulation of H_2_O_2_ which reached the highest at 36°C for 6 h. Furthermore, sensitivity of *P*. *ostreatus* mycelia to high concentration of H_2_O_2_ was significantly higher than that of *T*. *asperellum* mycelia. Therefore, it is shown that *T*. *asperellum* can produce H_2_O_2_ to inhibit and damage *P*. *ostreatus* mycelia in the process of mycoparasitism, and high temperature can further promote the accumulation of H_2_O_2_ to enhance the ability of inhibition.

To our knowledge, this study is the first to report the relationship between high temperature and rampant growth of *Trichoderma*. All present results show that high temperature is an important factor that enhances abilities of *T*. *asperellum* to infect *P*. *ostreatus*. This study provides theoretical basis for comprehensive understanding of the outbreak of green mold disease.

## Supporting information

S1 Fig Infection of *T*. *asperellum* on *P*. *ostreatus* mycelia. **a** *P*. *ostreatus* bags were incubated at 28°C for 10 days. Then, *T*. *asperellum* discs were inoculated into bags and incubated at 28°C. *P*. *ostreatus* mycelia were not infected by *T*. *asperellum*. **b** *P*. *ostreatus* bags treated with 36°C for 2 days were infected by *T*. *asperellum* and covered with green conidia.
(TIF)

S2 Fig Mycelial growth rate on different concentrations of H_2_O_2_. *Trichoderma asperellum* and *P*. *ostreatus* mycelial discs were inoculated in the center of plates with different concentrations of H_2_O_2_. T: mycelial growth rate of *T*. *asperellum*. P: mycelial growth rate of *P*. *ostreatus*. Data were analyzed by Duncan’s ANOVA test. Error bars represent the standard deviation of three replicates.
(TIF)
