# Supplementary figures and images for: High temperature enhances the ability of Trichoderma asperellum to infect Pleurotus ostreatus mycelia
Source: PLoS One. 2017 Oct 26;12(10):e0187055. doi: 10.1371/journal.pone.0187055 (PMC5658199; doi:10.1371/journal.pone.0187055)

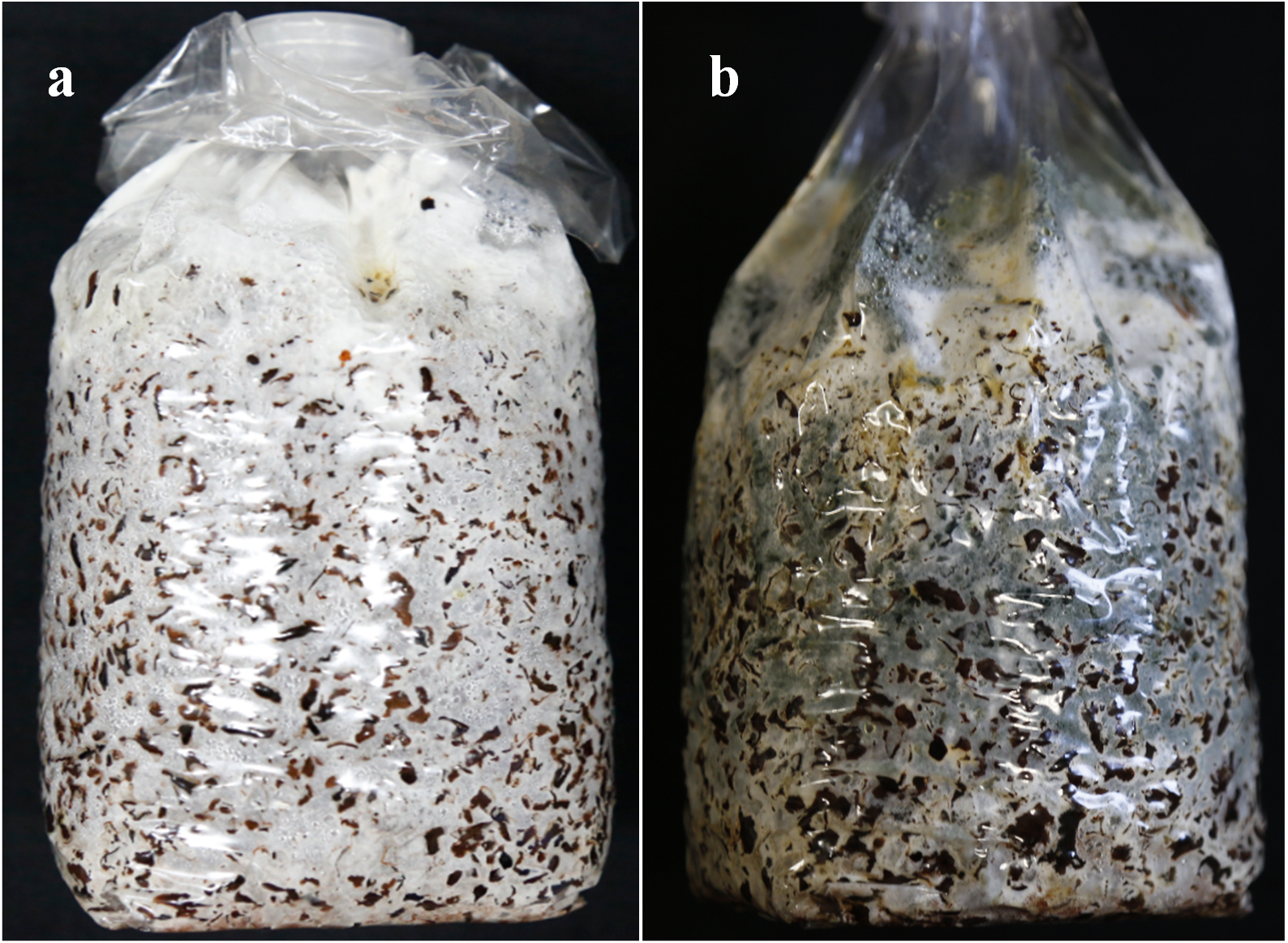

Supplement: S1 Fig — a P. ostreatus bags were incubated at 28°C for 10 days. Then, T. asperellum discs were inoculated into bags and incubated at 28°C. P. ostreatus mycelia were not infected by T. asperellum. b P. ostreatus bags treated with 36°C for 2 days were infected by T. asperellum and covered with green conidia. (TIF) [file pone.0187055.s001.tif]

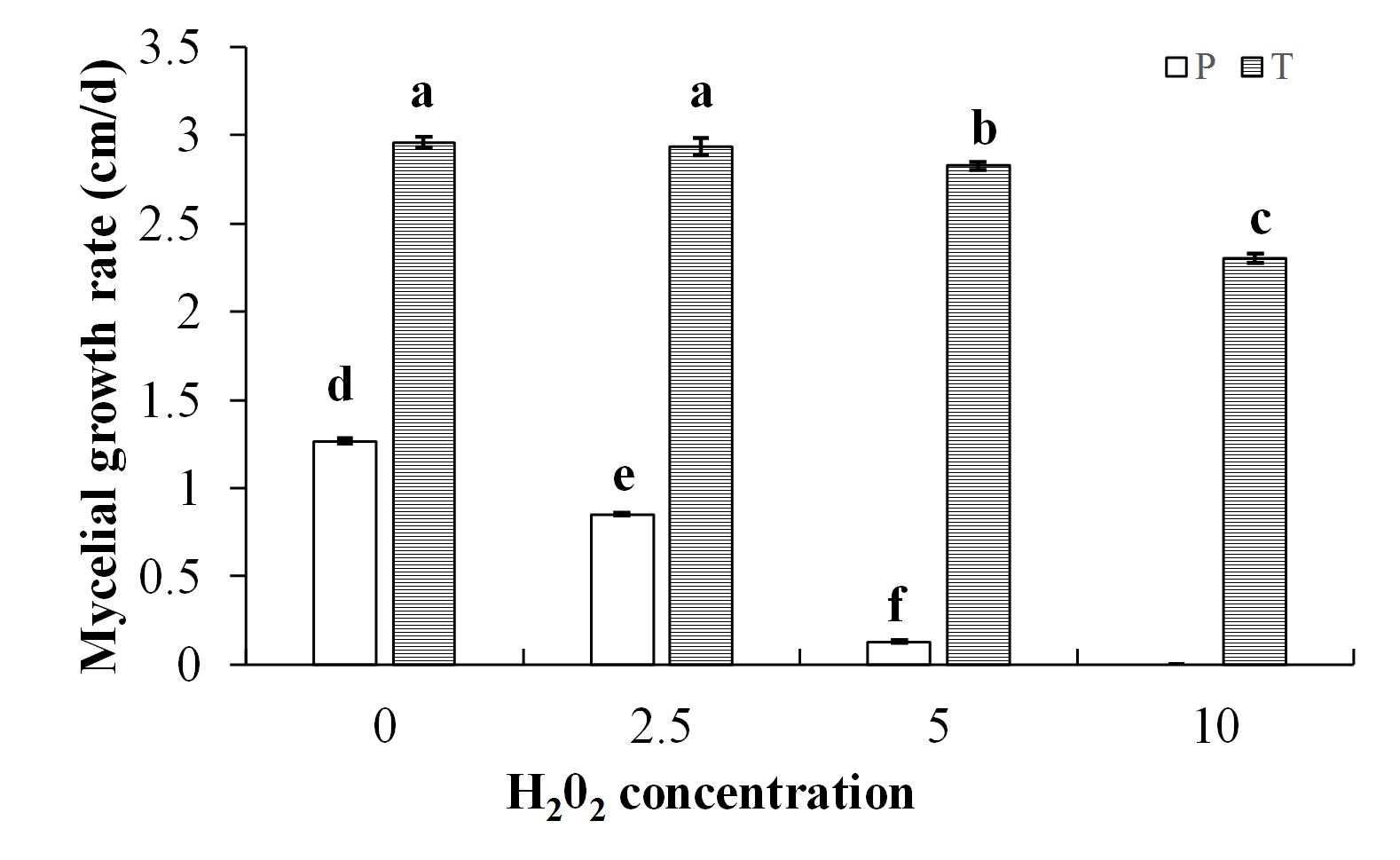

Supplement: S2 Fig — Trichoderma asperellum and P. ostreatus mycelial discs were inoculated in the center of plates with different concentrations of H2O2. T: mycelial growth rate of T. asperellum. P: mycelial growth rate of P. ostreatus. Data were analyzed by Duncan’s ANOVA test. Error bars represent the standard deviation of three replicates. (TIF) [file pone.0187055.s002.tif]
